# The Geographic Origins of Ethnic Groups in the Indian Subcontinent: Exploring Ancient Footprints with Y-DNA Haplogroups

**DOI:** 10.3389/fgene.2018.00004

**Published:** 2018-01-23

**Authors:** David G. Mahal, Ianis G. Matsoukas

**Affiliations:** ^1^School of Sport and Biomedical Sciences, University of Bolton, Bolton, United Kingdom; ^2^Extension Division, University of California, Los Angeles, Los Angeles, CA, United States

**Keywords:** Y chromosome, DNA, ethnic group, haplogroup, Indian subcontinent, human migration

## Abstract

Several studies have evaluated the movements of large populations to the Indian subcontinent; however, the ancient geographic origins of smaller ethnic communities are not clear. Although historians have attempted to identify the origins of some ethnic groups, the evidence is typically anecdotal and based upon what others have written before. In this study, recent developments in DNA science were assessed to provide a contemporary perspective by analyzing the Y chromosome haplogroups of some key ethnic groups and tracing their ancient geographical origins from genetic markers on the Y-DNA haplogroup tree. A total of 2,504 Y-DNA haplotypes, representing 50 different ethnic groups in the Indian subcontinent, were analyzed. The results identified 14 different haplogroups with 14 geographic origins for these people. Moreover, every ethnic group had representation in more than one haplogroup, indicating multiple geographic origins for these communities. The results also showed that despite their varied languages and cultural differences, most ethnic groups shared some common ancestors because of admixture in the past. These findings provide new insights into the ancient geographic origins of ethnic groups in the Indian subcontinent. With about 2,000 other ethnic groups and tribes in the region, it is expected that more scientific discoveries will follow, providing insights into how, from where, and when the ancestors of these people arrived in the subcontinent to create so many different communities.

## Introduction

### First arrivals

*Homo sapiens* or modern humans spread from Africa to Asia and Europe in several migratory movements (Stringer, 2000; Walter et al., 2000). Based on the geographical distances between populations and measures of population differentiation derived from quantitative cranial datasets, multiple dispersals took place between ~37 and 135 kya (1000 years ago) (Reyes-Centeno et al., 2015). The initial migrants traveled north and crossed into the Arabian Peninsula. Early archeological evidence of *H. sapiens* fossils outside Africa was discovered in the prehistoric caves of Qafzeh and Skhul, in present-day Israel. New mass-spectrometric techniques have dated these fossils to ~80–106 kya (McDermott et al., 1993). Some traveled further north into central Asia, which became the staging ground for migrations into Serbia and Europe.

The Indian subcontinent-comprising India, Pakistan, Bangladesh, Sri Lanka, Nepal, Bhutan, and Myanmar–became one of the first geographical regions of the world to be populated by *H. sapiens* (Dennell and Petraglia, 2012; Blinkhorn et al., 2013). One group from the Arabian Peninsula took the coastal route through India, Myanmar, and Malaysia to Australia. A study (Elhaik et al., 2013) conducted by the National Geographic Society's Genographic Project (Behar et al., 2007) found that people living in a village near Madurai in South India carried the same rare genetic markers as some Australian aborigines and people living in Africa (Wells, 2007). The findings showed a link between the three continents and confirmed that the people in Australia and India with this genetic marker were likely descendants of the original coastal migrants from Africa. More migrations out of Africa followed.

The migration through India was interrupted about 75 kya by the eruption of Mount Toba in Sumatra, Indonesia, which is recorded as one of the largest volcanic eruptions in this planet's history (Blinkhorn et al., 2014), resulting in an extended nuclear winter and ice age (Rampino and Self, 1993; Huang et al., 2001; Robock et al., 2009). Michael Petraglia and his team of archeologists discovered stone tools at Jwalapuram in Andhra Pradesh, South India, above and below a thick layer of ash from the Toba eruption (Petraglia et al., 2007). These tools match those used in Africa from the same period and suggested the presence of modern humans in India at the time of the Toba event. A recent theory refutes this research and contends that these were prehuman species (Mellars et al., 2013). After warming of the climate, new migrations out of Africa from ~50 kya populated India with large numbers of humans who later became known as Dravidians.

### Archaeological and historical perspectives

A recent archeological discovery was the *Homo sapiens balangodensis* (Balangoda man) in Sri Lanka, dated to ~37 kya (Tan, 2012). Other findings include the prehistoric rock shelters of Bhimbetka near Bhopal in Madhya Pradesh, India, which date back to 30,000 BCE. These rock shelters served as habitation sites during the lower Paleolithic period (Anon, 2017). There are more than 700 caves with more than 400 paintings carved in stone, which makes them one of the oldest known rock art sites in the world. Other archeological discoveries include the sites of the Indus Valley civilization in northwest India and Pakistan, which are dated ~8–9 kya (Khandekar, 2012). A recent discovery of one of the largest Indus Valley sites was made in Rakhighari, which is located about 160 km from New Delhi, and is dated ~7 kya (Subramanian and Khan, 2016). The people of the Indus Valley were known as the earliest agriculturists in South Asia (Harris, 1996).

Because language is a recent development, there is no written record of *ancient* Indian history. There is no reliable written history of the Indian subcontinent before Alexander the Great's campaign of India in 327 BCE (Smith, 1921). As a result, the deep ancient origins of the founder populations of the Indian subcontinent have remained ambiguous for a long time. In recent times, a series of migrations and invasions—both peaceful and violent—from adjoining areas became a recurring theme in the history of the Indian subcontinent. The different ethnicities of people that arrived and settled included Afghans, Arabs, Armenians, Aryans, Chinese, Greeks, Huns, Iranians, Mongols, Persians, Scythians, Syrians, Tajiks, Turks, Uzbeks, and others (Ahloowalia, 2009). The subcontinent has been aptly described as an “ethnological museum.”

### Tracing human origins

Y chromosome (Y-DNA) and mitochondrial DNA (MT-DNA) studies have been used to support ideas about modern human origins. These DNA technologies exploit two types of genetic markers: the short tandem repeats (STRs), and single nucleotide polymorphisms (SNPs). The STRs are found on the Y chromosome (Y-STRs) and used exclusively for tracing male lines of heredity. The SNPs are found on the Y chromosome and in MT-DNA. They are used to trace male and female lines of heredity. The result of the test is a set of numbers, referred to as the *haplotype*, that represents the allele values of DYS markers (D for DNA, Y for chromosome, and S for segment) on a portion of the DNA. The haplotype is used to identify the *haplogroup* of an individual. Thus, the *haplogroup* represents a group of people who have inherited common genetic characteristics from the same most recent common ancestor (MRCA) going back several thousand years. All humans belong to haplogroups which are designated according to their Y-DNA and MT-DNA.

The nonrecombining portion of the human Y chromosome is paternally inherited. This chromosome passes from father to son and is essentially unchanged; however, occasionally random small changes, known as polymorphisms, occur. These polymorphisms serve as beacons or markers and can be mapped. Correct interpretation of these changes in the Y chromosome can improve our understanding of temporal and spatial aspects of human history. Thus, the Y chromosome haplogroup, which is a population group descended from the MRCA, can be used as a valuable tool to trace the paternal line of the individual (Jobling and Tyler-Smith, 2003).

Y-DNA tests are available only for men. Short tandem repeats (STRs) or single nucleotide polymorphisms (SNPs) on the Y chromosome are assessed. Because Y-DNA haplogroups are closely linked to geography and populations, they are important genetic indicators to trace paternal lineages and their ancient origins. This study has relied on the Y-DNA *haplogroup*, as the primary gauge for exploring deep ancestry and geographical origins of the MRCAs.

Recent developments in DNA science were assessed to provide a contemporary perspective of the ancient geographic origins of 50 key ethnic groups of the Indian subcontinent. After identifying the Y haplogroups of these ethnic groups, the ancient geographical origins were ascertained from genetic markers in the Y-DNA Haplogroup Tree and published sources. The ancient origins of the ethnic groups were traced to 14 different geographical areas of this world. A startling new assessment of the genetic ancient origins of the ethnic groups was revealed with DNA science.

## Materials and methods

### Sample dataset

A dataset of 2,504 Y chromosome profiles of 50 ethnic groups in the Indian subcontinent was compiled from eight different sources (Table 1). These included the Genographic Project database (Behar et al., 2007; Genographic, 2016), with permission of the National Geographic Society, and seven published sources (Sengupta et al., 2006; Nagy et al., 2007; Zhao et al., 2008; Giroti and Talwar, 2010; Nair et al., 2011; Chennakrishnaiah et al., 2013; Lee et al., 2014). The dataset represented 50 geographically diversified ethnic groups of the subcontinent, of which 39 groups were in India, nine were in Pakistan, and two were in Bangladesh.

**Table 1:** Datasets used in this study.

**Dataset**	**Details**	**References**
The National Geographic Society's Genographic Project	The Genographic Project is studying the genetic signatures of ancient human migrations and creating a database of yDNA and mtDNA profiles. Currently, there are over 800,000 participants from over 140 countries.	Genographic, 2016
The Ethnic Groups of South Asia	The study covered a high-resolution assessment (69 informative Y-chromosome binary markers and 10 microsatellite markers) of a large set of representative ethnic groups of South Asia. This included 728 samples from India representing 36 populations, with 17 tribal populations, from six geographic regions and different social and linguistic categories, and 176 samples from Pakistan representing eight populations.	Sengupta et al., 2006
The Origin of Romanies	The haplotype frequencies for 11 Y-STR markers in a Romani population (*n* = 63) from Slovakia, Jats of Haryana (*n* = 84), and Jat Sikhs (*n* = 80) from India were assessed.	Nagy et al., 2007
Paternal Lineages among North Indians	A total of 32 Y-chromosomal markers in 560 North Indian males collected from three higher caste groups (Brahmins, Chaturvedis, and Bhargavas) and two Muslims groups (Shia and Sunni) were genotyped.	Zhao et al., 2008
The Himachal Pradesh, India Autosomal and Y-Chromosome Study	Genotypic analysis of 48 Malani individuals at 15 highly polymorphic autosomal STR loci.	Giroti and Talwar, 2010
The Ezhava Population of Kerala	Haplotype analysis of the Ezhava population of Kerala (*n* = 104), South India, using eight STR loci on the Y chromosome.	Nair et al., 2011
The Dravidian populations	Two Dravidian populations, namely Lingayat (*n* = 101) and Vokkaliga (*n* = 102), who represent the two major communities of the Karnataka state, were examined using high-resolution analyses of Y-SNPs and 17 Y-STR loci.	Chennakrishnaiah et al., 2013
The Pathans Group of Pakistan	Haplotype analysis of 22 Y-STR haplotypes and Y haplogroup distribution in Pathans (*n* = 270) of Pakistan.	Lee et al., 2014

*A master dataset of 2,504 Y-chromosome profiles of 50 ethnic groups in the Indian subcontinent was compiled from eight different sources*.

The haplogroups of 2,191 or 88% of the profiles in the dataset were predetermined at source based on examination of SNPs on the Y chromosome with actual DNA samples of men. These were collected from the Genographic Project database and published sources. For the remaining 313, or about 12% of the profiles in the dataset, the allele frequencies were collected from published material, and the haplogroups were identified with Whit Athey's Haplogroup Predictor software (Athey, 2006).

All haplogroups—those predetermined at source and identified with the software—were merged and sorted in a database according to their ethnic groups. Only the predominant top-level haplogroups were identified (the subclades or subhaplogroups were not used).

### Haplogroup prediction software

Several software programs are available that use mathematical calculations to predict haplogroups from Y-STR profiles. A study of a software tool, Haplogroup Classifier, developed at the University of Arizona showed that by using machine learning algorithms and data derived from a set of Y-linked STRs, it was possible to assign Y chromosome haplogroups to individual samples with a high degree of accuracy (Schlecht et al., 2008). Another software tool, yHaplo, was developed at 23andMe, a DNA testing company, to enable researchers to identify the Y chromosome haplogroups of men in genetic samples. The software has been tested on more than 600,000 samples of men in the 23andMe database (Poznik, 2016). For this study, Whit Athey's online Haplogroup Predictor software (http://www.hprg.com/hapest5/) that utilizes Y-STR values with a Bayesian-allele frequency approach (Athey, 2005, 2006) was exploited.

Whit Athey's software offers fast and easy prediction of a Y chromosome haplogroup from Y-STR values. The latest version of the program (10 Dec 2012) has adopted the 111-marker set of Family Tree DNA as the standard. Of the 86 markers used in the previous version of the program, only DYS508 is not included in the 111-marker set. A “batch” version of the software is now available for application to large numbers of haplotypes.

The Bayesian approach used in the software considers the frequency of each haplogroup in the geographic region where the haplotype originated. These frequencies are called the “prior probabilities” or “priors,” and they are different from one geographical area to another (for example, Northwest Europe and South Asia). The software provides an option to select the desired geographical area for the analyses. The current options available are Northwest Europe, East Europe, Mediterranean, and Equal Priors. After the area selection is made, the markers are entered in an online form. The results provide “goodness-of-fit” scores for haplogroups, and the probabilities for each score. If a haplogroup gets a probability of 100%, it means that the haplotype most likely only exists in that haplogroup. Typically, the results produce more than one haplogroup for a haplotype. For this study, 9–17 Y-STR markers were used for each profile, and the haplogroup with the highest probability was selected. The software was deployed for only 313 profiles in the total dataset.

The software examined up to fourteen Y-STR loci (DYS19, DYS385a, DYS385b, DYS389I, DYS389II, DYS390, DYS391, DYS392, DYS393, DYS437, DYS438, DYS439, DYS448, DYS456, DYS458) for these profiles. The allele DNA sequence variants of these markers are explained in Table 2.

**Table 2:** The allele sequence variants that have been exploited in this study.

**Y-STR marker**	**Description**	**DNA sequence repeat motif**	**Allele range**	**Mutation rate (%)**	**GenBank Accession**	**Aliases**
DYS19	n/a	[TAGA]	10–19	0.25	X77751; *n* = 9 repeats AC017019; *n* = 12 repeats	DY-27H39; DYS394
DYS385a	The order of DYS385a may be reversed. Its sequence is referred to as the Kittler order.	[GAAA]	7–28	0.21	AC022486; *n* = 11 repeats Z93950; has 10 repeats	DYS385 I
DYS385b	The order of DYS385b may be reversed. Its sequence is referred to as the Kittler order.	[GAAA]_n_	7–28	0.21	AC022486; *n* = 11 repeats Z93950; has 10 repeats	DYS385 II
DYS389I	DYS389 is a multi-copy marker, and includes DYS389i and DYS389ii. DYS389ii refers to the total length of DYS389. Therefore, when there is a one-step mutation at DYS389i, it will also appear in DYS389ii.	[TCTG]_3_ [TCTA]	9–17	0.24	AC011289 AF140635	DYS389a
DYS389II	DYS389 is a multi-copy marker, and includes DYS389i and DYS389ii. DYS389ii refers to the total length of DYS389. Therefore, when there is a one-step mutation at DYS389i, it will also appear in DYS389ii.	[TCTG]_n_[TCTA]pN48[TCTG]_3_[TCTA]_q_	24–34	0.35	AC011289 AF140635	DYS389b
DYS390	n/a	[TCTG]_8_[TCTA]_n_[TCTG]_1_[TCTA]_4_	17–28	0.25	AC011289; *n* = 8 repeats, *m* = 11 repeats, *p* = 1 repeat, *q* = 4 repeats	n/a
DYS391	n/a	[TCTA]_n_	6–14	0.28	G09613; *n* = 11 repeats AC011302; *n* = 11 repeats	n/a
DYS392	n/a	[TAT]n	6–17	0.07	G09867; *n* = 16 repeats AC06152; *n* = 13 repeats	n/a
DYS393	n/a	[AGAT]_n_	9–17	0.08	G09601; *n* = 13 repeats AC006152; *n* = 12 repeats	DYS395
DYS437	n/a	[TCTA]_n_[TCTG]_2_[TCTA]_4_	13–17	0.13	AC002992; *n* = 10 repeats	
DYS438	n/a	[TTTTC]_n_	6–14	0.07	AC002531; *n* = 10 repeats	
DYS439	n/a	[GATA]_n_	9–14	0.61	AC002992; has 13 repeats	
DYS448	n/a	[AGAGAT]_n_N42[AGAGAT]_m_	20–26	0.11	AC025227; 22 repeats	
DYS456	n/a	[AGAT]_n_	13–18	0.53	AC010106.2	
DYS458	n/a	[GAAA]_n_	13–20	1.06	AC010902	

*n, p, and q represent number of individual repeats per short tandem repeat unit. Reference motifs are based on sequences provided in STRBase (http://www.cstl.nist.gov/strbase/ystr_fact.htm). Mutation rates are those per generation, as estimated in Chandler (2006)*.

### The Y-DNA haplogroup tree

The geographic origins of a Y chromosome haplogroup for males can be deciphered from the phylogenetic tree of mankind, or the Y-DNA Haplogroup Tree, maintained by the International Society of Genetic Genealogy (ISOGG, 2016). The haplogroups contain many branches called subhaplogroups or subclades. The top-level haplogroups are expressed as letters (A, B, C, etc.). Their subhaplogroups or subclades are expressed as letters and numbers (G2, R1b1, E3b1b, etc.). The markers on the phylogenetic tree provide pieces of evidence regarding the date and geographical origin of the MRCA in the distant past. The geographic origins of the 14 haplogroups identified from the dataset were deciphered from the phylogenetic tree and other published sources.

## Results

The data revealed that 14 different haplogroups representing 14 different geographic origins were present in the 50 ethnic groups used in this study (Table 3), confirming multiple lines of ancestry and geographic origins. Every ethnic group had members that belonged to more than one haplogroup, indicating that they had different lines of ancestors. There was no ethnic group in these analyses that could trace the genetic ancestry of all its members to a single MRCA. For example, members of the large Brahmin ethnic group belonged to 11 different haplogroups, indicating 11 different lines of ancestors. Similarly, the Malayali and Nair groups had members in 10 different haplogroups, indicating at least 10 different ancestral lines.

**Table 3:** Fifty ethnic groups of the Indian Subcontinent (*n* = 2,504) represented in 14 Y-Chromosome Haplogroups, with their markers and date scale.

**Markers**		**M130**	**M96**	**M89**	**M201**	**M69**	**M170**	**M304**	**M9**	**M11**	**M175**	**M48**	**M242**	**M207**	**M184**	
**Date scale, kya**		~**50**	~**30–40**	~**45**	~**10–23**	~**30**	~**25**	~**15**	~**40**	~**25–30**	~**35**	~**35**	~**15–20**	~**4–27**	~**25**	
**Ethnic groups**	**n**	**C**	**E**	**F**	**G**	**H**	**I**	**J**	**K**	**L**	**O**	**P**	**Q**	**R**	**T**	**Data sources (8)**
Bangladesh, Bangladeshi	22					√		√			√			√		Genographic, 2016
Bangladesh, Bengali	49	√			√	√		√		√	√		√	√		Genographic, 2016
India, Agharia	10					√		√						√		Sengupta et al., 2006
India, Ambalakarar	29			√	√	√		√		√				√		Sengupta et al., 2006
India, Assamese	6					√					√		√	√		Genographic, 2016
India, Bhargavas	96	√		√		√		√	√		√	√		√		Zhao et al., 2008
India, Brahmin	152	√	√	√	√	√		√	√	√	√	√		√		Sengupta et al., 2006; Zhao et al., 2008; Nair et al., 2011
India, Chamar	18	√				√								√		Sengupta et al., 2006
India, Chaturvedis	88	√		√		√		√	√	√	√	√		√		Zhao et al., 2008
India, Ezhava	113		√		√	√	√	√		√			√	√	√	Nair et al., 2011; Genographic, 2016
India, Gujarati	116	√		√		√		√		√			√	√		Genographic, 2016
India, Halba	20			√		√					√			√		Sengupta et al., 2006
India, Ho	30					√					√					Sengupta et al., 2006
India, Irula	30	√		√		√		√		√				√		Sengupta et al., 2006
India, Iyengar	43	√			√	√		√		√			√	√		Sengupta et al., 2006; Genographic, 2016
India, Iyer	51	√			√	√		√		√			√	√		Sengupta et al., 2006; Genographic, 2016
India, Jamatia	30					√					√			√		Sengupta et al., 2006
India, Jat Haryana	108				√	√	√	√		√			√	√	√	Nagy et al., 2007; Genographic, 2016
India, Jat Sikh	98		√		√	√	√	√		√			√	√		Nagy et al., 2007; Genographic, 2016
India, Kamar	30			√		√				√	√					Sengupta et al., 2006
India, Kashmiri	21					√		√		√				√		Genographic, 2016
India, Koknasth Brahmin	25					√		√		√				√		Sengupta et al., 2006
India, Konda Reddy	30			√		√					√			√		Sengupta et al., 2006
India, Konkane	53	√		√	√	√		√		√			√	√	√	Genographic, 2016
India, Kota	15			√		√								√		Sengupta et al., 2006
India, Koya Dora	27			√		√		√			√					Sengupta et al., 2006
India, Kurumba	19			√		√				√				√		Sengupta et al., 2006
India, Lingayat	101	√		√	√	√		√		√				√		Chennakrishnaiah et al., 2013
India, Lodha	20	√				√		√						√		Sengupta et al., 2006
India, Malani	30					√		√		√				√		Giroti and Talwar, 2010
India, Malayali	97	√		√	√	√	√	√		√			√	√	√	Genographic, 2016
India, Maratha	88	√		√	√	√		√		√			√	√		Sengupta et al., 2006; Genographic, 2016
India, Mizo	27					√					√					Sengupta et al., 2006
India, Muria	20			√		√					√					Sengupta et al., 2006
India, Nair	48	√	√		√	√	√	√		√			√	√	√	Nair et al., 2011; Genographic, 2016
India, Pallan	29	√		√		√		√		√				√		Sengupta et al., 2006
India, Rajput	47	√		√		√		√		√	√			√		Genographic, 2016
India, Tripuri	20										√			√		Sengupta et al., 2006
India, Vanniyar	25	√		√		√		√		√				√		Sengupta et al., 2006
India, Vellalar	32		√			√	√	√		√				√		Sengupta et al., 2006; Nair et al., 2011
India, Vokkaliga	102	√		√	√	√		√		√	√			√		Chennakrishnaiah et al., 2013
Pakistan, Balochi	29		√			√		√		√				√		Sengupta et al., 2006; Genographic, 2016
Pakistan, Brahui	25	√			√	√		√		√	√			√		Sengupta et al., 2006
Pakistan, Burusho	20	√			√	√		√	√	√	√			√		Sengupta et al., 2006
Pakistan, Hazara	27	√					√	√			√		√	√		Sengupta et al., 2006
Pakistan, Kalash	20				√	√		√		√				√		Sengupta et al., 2006
Pakistan, Makrani	20		√					√		√			√	√		Sengupta et al., 2006
Pakistan, Pashtun	20					√		√		√			√	√		Genographic, 2016
Pakistan, Pathan	288	√			√	√		√	√	√	√		√	√	√	Sengupta et al., 2006; Lee et al., 2014
Pakistan, Sindhi	40		√		√	√		√		√			√	√		Sengupta et al., 2006; Genographic, 2016
Total	2,504	76	14	71	84	403	15	279	37	281	175	11	65	963	30	
Percent (%)	100	3.0	0.6	2.8	3.4	16.1	0.6	11.1	1.5	11.2	7.0	0.4	2.6	38.5	1.2	

*Sources for markers and date scale: (Smolenyak and Turner, 2004; Wells, 2007; Y-DNA Haplogroup Tree, markers, and descriptions at ISOGG, http://isogg.org/tree/index.html). kya = 1000 years ago*.

Some groups had few ancestral lines. The tribal Ho and Mizo groups had members in only two haplogroups, with O being the predominant one, indicating that there may be one major line of ancestry. Similarly, the Malanis, who live in the small hermit village of Malana in the Himalayas with a population of only about 1,100 people, primarily belonged to only two predominant haplogroups, J and R (Giroti and Talwar, 2010).

Although there were 14 haplogroups in the total dataset, about 90% of the people belonged to seven haplogroups: F, G, H, J, L, O, and R. About 77% of the people belonged to the four largest haplogroups R, H, L, and J.

These haplogroups are described below.

### Haplogroup R (38.5%)

This is one of the largest haplogroups in India and Pakistan. This is also the largest haplogroup in the dataset used in this study. It originated in north Asia about 27,000 years ago (ISOGG, 2016). It is one of the most common haplogroups in Europe, with its branches reaching 80 percent of the population in some regions (Eupedia, 2017). One branch is believed to have originated in the Kurgan culture, known to be the first speakers of the Indo-European languages and responsible for the domestication of the horse (Smolenyak and Turner, 2004). From somewhere in central Asia, some descendants of the man carrying the M207 mutation on the Y chromosome headed south to arrive in India about 10,000 years ago (Wells, 2007).

### Haplogroup H (16.1%)

This is an old haplogroup with a large representation in the Indian subcontinent. It can be referred to as the Indian haplogroup. Originally from the Middle East or south central Asia, marker M69 originated in western India about 30000 years ago (Wells, 2007). This group is considered part of a second wave of migrations to the Indian subcontinent. The Romany people, also known as gypsies and believed to originate from India, belong to a subclade of this haplogroup (ISOGG, 2008).

### Haplogroup L (11.2%)

This haplogroup is present in the Indian population at an overall frequency of about 7–15% (Basu et al., 2003; Cordaux et al., 2004). Genetic studies indicate that this may be one of the original haplogroups of the creators of Indus Valley Civilization (McElreavey and Quintana-Murci, 2005; Sengupta et al., 2006). It has a frequency of about 28% in western Pakistan and Baluchistan, from where the agricultural creators of this civilization emerged (Qamar et al., 2002). The origins of this haplogroup can be traced to marker M11, and the rugged and mountainous Pamir Knot region in Tajikistan (Wells, 2007), which is also the home of the Bactria-Margiana Archaeological Complex that represents the Oxus civilization of around 4000 BCE (Wood, 2007).

### Haplogroup J (11.1%)

The ancestor carrying the M304 mutation was born around 15000 years ago in the Middle East area known as the Fertile Crescent, comprising Israel, the West Bank, Jordon, Lebanon, Syria, and Iraq. There is a dominant Arabic lineage. This group and its subclades are found predominantly around the coast of the Mediterranean, the Middle East, North Africa, and Ethiopia. Middle Eastern traders brought this genetic marker to the Indian subcontinent (Kerchner, 2013).

### Geographic origins

**Figure 1:**
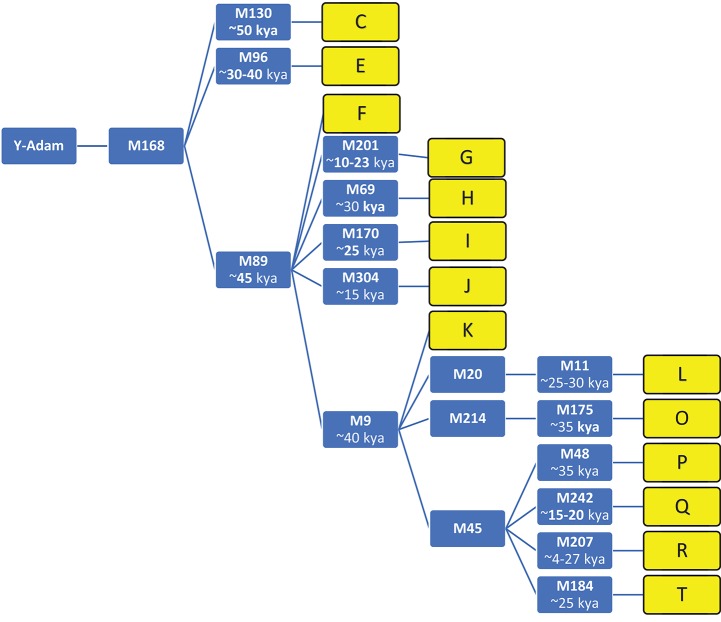
Fourteen top level haplogroups, and recent markers with date scale. (Smolenyak and Turner, 2004; Wells, 2007); Y-DNA Haplogroup Tree, markers, and descriptions at ISOGG, http://isogg.org/tree/index.html), kya, thousand years ago.

A phylogenetic tree (without branches) showing the top-level Y-DNA haplogroups and markers of the 14 ethnic groups used in this study appears in Figure 1.

The geographic origins of the 14 different haplogroups were ascertained from the phylogenetic tree of mankind maintained by the International Society of Genetic Genealogy (ISOGG, 2016), and published sources. They are summarized in Table 4.

**Table 4:** Ancient geographic origins of 14 Y-chromosome haplogroups.

**Haplogroup**	**Marker**	**Age**	**Geographic origins**
C	M130	~50 kya	Southern Asia, part of first migration out of Africa, coastal India to Southeast Asia
E	M96	~30–40 kya	Northeast Africa, part of second migration out of Africa, initially settled in Middle East
F	M89	~45 kya	Northeastern Africa or the Middle East (in 90% of all non-African men), parent of HG's G–T
G	M201	~10–23 kya	Eastern edge of the Middle East, close to Himalayan foothills, Indus Valley
H	M69	~30 kya	South central Asia, known as the “Indian Marker”
I	M170	~25 kya	Europe, Near East, Central Asia, known as the “European haplogroup”
J	M304	~15 kya	Fertile Crescent (Mesopotamia, the land in and around the Tigris and Euphrates rivers)
K	M9	~40 kya	Iran or south-central Asia, diverged from the M89 Middle Eastern clan
L	M11	~25–30 kya	Pamir Knot region (Hindu Kush, Tian Shan, Himalayas) in Tajikistan
O	M175	~35 kya	Central or East Asia, part of M9 Eurasian clan, early Siberians
P	M48	~35 kya	Central Asia, part of M9 Eurasian clan, north of Hindu Kush mountains
Q	M242	~15–20 kya	Siberia (North Asia), descendants first arrivals in North America
R	M207	~4–27 kya	Central Asia (from the Caspian Sea to border of western China)
T	M184	~25 kya	Low frequencies Europe, the Middle East, North Africa, and East Africa

*Smolenyak and Turner, 2004; Wells, 2007; Y-DNA Haplogroup Tree, markers, and descriptions at ISOGG, http://isogg.org/tree/index.html. kya = 1000 years ago*.

## Discussion

### Sample size

In statistical analyses, as the population increases in size, the sample size increases at a diminishing rate, and remains relatively constant when it reaches a size of 380 or more. At about 384, the sample is generally representative for a population of one million, or more (Krejcie and Morgan, 1970). Therefore, to ascertain a representative distribution of haplogroups in any large ethnic community, ideally the sample size should be 380, and preferably larger.

The samples available for the 50 ethnic groups used in this study were less than the ideal size, and ranged from 6 for the Assamese in India to 288 for the Pathans in Pakistan. Although the sample for each ethnic group revealed key haplogroups, it did not represent a statistically significant distribution for the total population of the ethnic group. Larger samples for these ethnic groups are likely to reveal a few additional haplogroups and provide a more complete picture for each ethnic group.

### Potential errors in identifying haplogroups

Because of the need for precision in matters relating to criminal and civil laws, the forensic genetics community is generally not in favor of determining haplogroups with STR profiles. It is held that STR haplotypes are not always identical by descent, but also identical by state, and can be rooted in different haplogroups.

A study that used STR profiles of 119 males in Argentina to determine haplogroups with two software programs—Whit Athey's Haplogroup Predictor (used in this study), and a Haplogroup Classifier developed at the University of Arizona—showed that the results were not totally accurate (Muzzio et al., 2011). Another study of 165 males in Nicaragua showed that Athey's Haplogroup Predictor produced accurate results for 95.2% of the sample, but 4.8% of the results were inaccurate (Núñez et al., 2012). For greater reliability in identifying Y chromosomal haplogroups, the forensic community's preferred method is to analyze single nucleotide polymorphisms (SNPs) on the Y chromosome in the lab with actual DNA samples.

Athey has explained that the main drawback of the haplogroup prediction method in his software is the size of the database of some Y-STR haplotypes from which the allele frequencies are calculated. For most haplogroups there is sufficient Y-STR haplotype data. However, for some haplogroups, such as C, H, L, N, and Q, the database of Y-STR haplotypes is smaller, and the results may be prone to error (Athey, 2006).

From the total dataset used in this study, 313 or about 12% of the records were processed through Athey's software to determine their haplogroups. Assuming an error rate of 5%, as reported in the Nicaraguan study (Núñez et al., 2012), 16 haplotypes (5% of 313) may have identified incorrect haplogroups. That represents a potential error of only 0.6% (16/2504) in the total dataset used in the study.

### Population mixture

The population of the Indian subcontinent at ~12 kya was statistically estimated at about 100,000 people (McEvedy and Jones, 1979). Currently, there are about 1.7 billion people on this subcontinent. The increase in population reflects a complex history of migrations and invasions of people from outside the subcontinent, resulting in an influx of foreign genes to the subcontinent.

When the population of the subcontinent was small, people did not travel far. However, over time, humans have moved to new and distant habitats. They have also shown variations in their post-marital residence practices. About 70% of humans practice some form of patrilocality, with men remaining in and women migrating from their household, clan, lineage, tribe, or village (Fox et al., 1967; Murdoch, 1981). Some societies display matrilocal or bilocal migration patterns, with men and members of both sexes leaving their birthplace to live with their mate elsewhere (Fox et al., 1967). It is believed these practices prevailed in the Indian subcontinent in ancient times. The people admixed freely and gradually scattered in different directions, merging with communities they found in their paths, or creating entirely new communities.

### Emergence of endogamy

Genetic studies have shown that most ethnic groups of the Indian subcontinent descended from a mixture of two divergent populations. These were Ancestral North Indians related to Central Asians, Middle Easterners, Caucasians, and Europeans and Ancestral South Indians who were not closely related to any groups outside the subcontinent (Reich et al., 2009; Moorjani et al., 2013). After the arrival of Indo-European speakers in North India about 4 kya, the caste system was introduced, and a stratified social hierarchy evolved.

The upper-caste populations were thought to have started practicing and encouraging endogamy about 70 generations (more than 2,000 years) ago (Basu et al., 2016). Another study suggested that endogamy originated much later, around the time of foreign invasions in north India (Vadivelu, 2016). As ethnic groups developed with their own identities, endogamy in the Indian subcontinent became the general norm.

The preference for close kin unions, i.e., consanguineous marriages between people, such as cousins, who have at least one recent common ancestor, is another type of endogamy. Currently, couples related as second cousins or closer account for an estimated 10.4% of the global population, with the highest rates in certain regions including West, Central, and South Asia (Bittles and Black, 2010). According to the International Institute for Population Sciences in Mumbai, about 16% of marriages in India are consanguineous (Kuntla et al., 2013). In Pakistan, where first cousin marriages have occurred for generations, the rate is 67% (Yaqoob et al., 1993).

At this time, there are several thousand different ethnic and tribal groups in the Indian subcontinent (Papiha, 1996; Xing et al., 2010). Members of these communities share common self-identities that are based on languages, customs, cuisines, and at least six major religions. There are 22 official languages and hundreds of dialects in the country (Annamalai, 2006), which reflect the genetic diversity of the population. About 125 million people—roughly 10% of the population—now speak the English language (Mehtabul et al., 2013), and members of many ethnic groups have migrated to other countries throughout the world.

Although endogamy has become the general norm in the Indian subcontinent, and consanguinity is practiced in some communities, there has been considerable admixture in the past, resulting in a very mixed set of genes and geographic origins throughout the subcontinent. This is evidenced by the distribution of 14 different haplogroups in the 50 ethnic groups used in this study.

### Genetic distance

In another study, the results of AMOVA (analysis of molecular variance) and MDS (multidimensional scaling plots) tests confirmed that the ethnic group of Jats had genetic affinities with several foreign populations (Mahal and Matsoukas, 2017). Similar studies of other ethnic groups of the Indian subcontinent will provide additional insights into their genetic makeup and geographic origins.

## Conclusion

The human Y chromosome provides a powerful molecular tool for analyzing Y-STR haplotypes and determining their haplogroups, which in turn lead to the ancient geographic origins of individuals. For this study, 50 ethnic groups in the Indian subcontinent were analyzed, and their haplogroups were identified. Using markers from the Y-DNA haplogroup tree and available descriptions of haplogroups, the geographic origins and migratory paths of the ancestors were ascertained and documented.

The results showed that every ethnic group in the dataset had members that belonged to more than one haplogroup, indicating multiple lines of ancestry and geographic origins. Additionally, even with their potentially different languages, religions, nationalities, customs, cuisines, and physical differences, members of different ethnic groups who belonged to the same haplogroup were genetically related and had the same ancient MRCAs and geographic origins in the distant past. Although historians have attempted scholarship on the deep origins of people, their assessments do not go back far enough in time because of lack of documentation. Based on recent developments in DNA science, this study has provided new insights into the ancient multi-source geographic origins of some distinct ethnic groups of the Indian subcontinent. It is expected that more scientific studies will follow, providing insights about where and when the founding populations of the Indian subcontinent arrived, and how they spread in different directions to create so many diverse ethnic communities.

## Ethics statement

This study presented in the manuscript did not involve human or animal subjects. All data used in the study are from existing databases and published sources, which are cited.

## Author contributions

DM: analyzed data and wrote the paper; IM: wrote the paper.

### Conflict of interest statement

The authors declare that the research was conducted in the absence of any commercial or financial relationships that could be construed as a potential conflict of interest.

## References

[B1] Ahloowalia B. S. (2009). Invasion of the Genes: Genetic Heritage of India. New York, NY: Eloquent Books.

[B2] Annamalai E. (2006). India: language situation, in Encyclopedia of Language & Linguistics, ed Brown K. (Amsterdam: Elsevier), 610–613.

[B3] Anon (2017). World Heritage Sites - Rock Shelters of Bhimbetka. Available online at: http://asi.nic.in/asi_monu_whs_rockart_bhimbetka_detail.asp: Archaeological Survey of India, Government of India (Accessed January 10, 2017).

[B4] Athey T. W. (2005). Haplogroup prediction from Y-STR values using an allele frequency approach. J. Genet. Geneal. 1, 1–7.

[B5] Athey T. W. (2006). Haplogroup prediction from Y-STR values using a Bayesian-Allele frequency approach. J. Genet. Geneal. 2, 34–39.

[B6] Basu A. Mukherjee N. Roy S. Sengupta S. Banerjee S. Chakraborty M.. (2003). Ethnic India: a genomic view, with special reference to peopling and structure. Genome Res. 13, 2277–2290. 10.1101/gr.1413403 14525929 PMC403703

[B7] Basu A. Sarkar-Roy N. Majumder P. P. (2016). Genomic reconstruction of the history of extant populations of India reveals five distinct ancestral components and a complex structure. Proc. Natl. Acad. Sci. U.S.A. 113, 1594–1599. 10.1073/pnas.1513197113 26811443 PMC4760789

[B8] Behar D. M. Rosset S. Blue-Smith J. Balanovsky O. Tzur S. Comas D.. (2007). The genographic project public participation mitochondrial DNA database. PLoS Genet. 3:e104. 10.1371/journal.pgen.0030104 17604454 PMC1904368

[B9] Bittles A. H. Black M. L. (2010). Consanguineous marriage and human evolution. Annu. Rev. Anthropol. 39, 193–207. 10.1146/annurev.anthro.012809.105051

[B10] Blinkhorn J. Achyuthan H. Petraglia M. Ditchfield P. (2013). Middle Palaeolithic occupation in the Thar Desert during the upper Pleistocene: the signature of a modern human exit out of Africa? Quat. Sci. Rev. 77, 233–238. 10.1016/j.quascirev.2013.06.012

[B11] Blinkhorn J. Smith V. C. Achyuthan H. Shipton C. Jones S. C. Ditchfield P. D. (2014). Discovery of youngest Toba tuff localities in the Sagileru valley, south India, in association with Palaeolithic industries. Quat. Sci. Rev. 105, 239–243. 10.1016/j.quascirev.2014.09.029

[B12] Chandler J. F. (2006). Estimating per-locus mutation rates. J. Genet. Gen. 2, 27–33.

[B13] Chennakrishnaiah S. Perez D. Gayden T. Rivera L. Regueiro M. Herrera R. J. (2013). Indigenous and foreign Y-chromosomes characterize the Lingayat and Vokkaliga populations of southwest India. Gene 526, 96–106. 10.1016/j.gene.2013.04.074 23664983

[B14] Cordaux R. Aunger R. Bentley G. Nasidze I. Sirajuddin S. M. Stoneking M. (2004). Independent origins of Indian caste and tribal paternal lineages. Curr. Biol. 14, 231–235. 10.1016/j.cub.2004.01.024 14761656

[B15] Dennell R. Petraglia M. D. (2012). The dispersal of *Homo sapiens* across southern Asia: how early, how often, how complex? Quat. Sci. Rev. 47, 15–22. 10.1016/j.quascirev.2012.05.002

[B16] Elhaik E. Greenspan E. Staats S. Krahn T. Tyler-Smith C. Xue Y.. (2013). The GenoChip: a new tool for genetic anthropology. Genome Biol. Evol. 5, 1021–1031. 10.1093/gbe/evt066 23666864 PMC3673633

[B17] Eupedia (2017). Genetics, Haplogroup R. Available online at: http://www.eupedia.com/europe/Haplogroup_R1b_Y-DNA.shtml (Accessed March 2, 2017).

[B18] Fox R. Wake C. S. Needham R. (1967). The development of marriage and kinship. Man 2:644 10.2307/2799373

[B19] Genographic (2016). The National Geographic Society's Genographic Project. Washington, DC: The National Geographic Society Available online at: https://genographic.nationalgeographic.com (Accessed August 5, 2016).

[B20] Giroti R. Talwar I. (2010). The most ancient democracy in the world is a genetic isolate: an autosomal and Y-Chromosome study of the hermit village of Malana (Himachal Pradesh, India). Hum. Biol. 82, 123–141. 10.3378/027.082.0201 20649396

[B21] Harris D. R. (1996). The Origins and Spread of Agriculture and Pastoralism in Eurasia. New York, NY: Routledge.

[B22] Huang C.-Y. Zhao M. Wang C.-C. Wei G. (2001). Cooling of the South China Sea by the Toba Eruption and correlation with other climate proxies ~71,000 years ago. Geophys. Res. Lett. 28, 3915–3918. 10.1029/2000GL006113

[B23] ISOGG (2008). Y-DNA Haplogroup, H., and Its Subclades - 2008. Available online at: https://isogg.org/tree/2008/ISOGG_HapgrpH08.html (Accessed December 4, 2016).

[B24] ISOGG (2016). International Society of Genetic Geology: Y-DNA Haplogroup Tree. Available online at: http://isogg.org/tree/index.html (Accessed December 16, 2016).

[B25] Jobling M. A. Tyler-Smith C. (2003). The human Y chromosome: an evolutionary marker comes of age. Nat. Rev. Genet. 4, 598–612. 10.1038/nrg1124 12897772

[B26] Kerchner C. F. (2013). YDNA Haplogroup Descriptions & Information Links. Available online at: http://www.kerchner.com/haplogroups-ydna.htm (Accessed September 2, 2016).

[B27] Khandekar N. (2012). Indus Valley 2,000 Years Older than Thought. Hindustan Times.

[B28] Krejcie R. V. Morgan D. W. (1970). Determining sample size for research activities. Educ. Psychol. Meas. 30, 607–610. 10.1177/001316447003000308

[B29] Kuntla S. Goli S. Sekher T. V. Doshi R. (2013). Consanguineous marriages and their effects on pregnancy outcomes in India. Int. J. Sociol. Soc. Policy 33, 437–452. 10.1108/IJSSP-11-2012-0103

[B30] Lee E. Y. Shin K.-J. Rakha A. Sim J. E. Park M. J. Kim N. Y.. (2014). Analysis of 22 Y chromosomal STR haplotypes and Y haplogroup distribution in Pathans of Pakistan. Forensic Sci. Int. Genet. 11, 111–116. 10.1016/j.fsigen.2014.03.004 24709582

[B31] Mahal D. G. Matsoukas I. G. (2017). Y-STR haplogroup diversity in the Jat population reveals several different ancient origins. Front. Genet. 8:121. 10.3389/fgene.2017.00121 28979290 PMC5611447

[B32] McDermott F. Grün R. Stringer C. B. Hawkesworth C. J. (1993). Mass-spectrometric U-series dates for Israeli Neanderthal/early modern hominid sites. Nature 363, 252–255. 10.1038/363252a0 8387643

[B33] McElreavey K. Quintana-Murci L. (2005). A population genetics perspective of the Indus Valley through uniparentally-inherited markers. Ann. Hum. Biol. 32, 154–162. 10.1080/03014460500076223 16096211

[B34] McEvedy C. Jones R. (1979). Atlas of World Population History. Harmondsworth; Middlesex: Penguin Books, Ltd.

[B35] Mehtabul A. Chin A. Prakash N. (2013). The returns to English-language skills in India. Econ. Devel. Cult. Change 61, 335–367. 10.1086/668277

[B36] Mellars P. Gori K. C. Carr M. Soares P. A. Richards M. B. (2013). Genetic and archaeological perspectives on the initial modern human colonization of southern Asia. Proc. Natl. Acad. Sci. U.S.A. 110, 10699–10704. 10.1073/pnas.1306043110 23754394 PMC3696785

[B37] Moorjani P. Thangaraj K. Patterson N. Lipson M. Loh P.-R. Govindaraj P.. (2013). Genetic evidence for recent population mixture in India. Am. J. Hum. Genet. 93, 422–438. 10.1016/j.ajhg.2013.07.006 23932107 PMC3769933

[B38] Murdoch G. P. (1981). Atlas of World Cultures. Pittsburgh, PA: University of Pittsburgh Press.

[B39] Muzzio M. Santos M. R. Bailliet G. Ramallo V. Motti J. M. B. Camelo J. S. L. (2011). Software for Y-haplogroup predictions: a word of caution. Int. J. Legal Med. 125, 143–147. 10.1007/s00414-009-0404-1 20082090

[B40] Nagy M. Henke L. Henke J. Chatthopadhyay P. K. Völgyi A. Zalán A.. (2007). Searching for the origin of Romanies: Slovakian Romani, Jats of Haryana and Jat Sikhs Y-STR data in comparison with different Romani populations. Forensic Sci. Int. 169, 19–26. 10.1016/j.forsciint.2006.07.020 16950585

[B41] Nair P. S. Geetha A. Jagannath C. (2011). Y-short tandem repeat haplotype and paternal lineage of the Ezhava population of Kerala, south India. Croat. Med. J. 52, 344–350. 10.3325/cmj.2011.52.344 21674830 PMC3118723

[B42] Núñez C. Geppert M. Baeta M. Roewer L. Martínez-Jarreta B. (2012). Y chromosome haplogroup diversity in a Mestizo population of Nicaragua. Forensic Sci. Int. Genet. 6:e192-195. 10.1016/j.fsigen.2012.06.011 22770600

[B43] Papiha S. S. (1996). Genetic variation in India. Hum. Biol. 68, 607–628. 8908794

[B44] Petraglia M. Korisettar R. Boivin N. Clarkson C. Ditchfield P. Jones S.. (2007). Middle Paleolithic assemblages from the Indian subcontinent before and after the Toba super-eruption. Science 317, 114–116. 10.1126/science.1141564 17615356

[B45] Poznik G. D. (2016). Identifying Y-chromosome haplogroups in arbitrarily large samples of sequenced or genotyped men. bioRxiv. 10.1101/088716

[B46] Qamar R. Ayub Q. Mohyuddin A. Helgason A. Mazhar K. Mansoor A.. (2002). Y-Chromosomal DNA Variation in Pakistan. Am. J. Hum. Genet. 70, 1107–1124. 10.1086/339929 11898125 PMC447589

[B47] Rampino M. R. Self S. (1993). Climate-Volcanism Feedback and the Toba Eruption of ~74,000 Years Ago. Q. Res. 40, 269–280. 10.1006/qres.1993.1081

[B48] Reich D. Thangaraj K. Patterson N. Price A. L. Singh L. (2009). Reconstructing Indian population history. Nature 461, 489–494. 10.1038/nature08365 19779445 PMC2842210

[B49] Reyes-Centeno H. Hubbe M. Hanihara T. Stringer C. Harvati K. (2015). Testing modern human out-of-Africa dispersal models and implications for modern human origins. J. Hum. Evol. 87, 95–106. 10.1016/j.jhevol.2015.06.008 26164107

[B50] Robock A. Ammann C. M. Oman L. Shindell D. Levis S. Stenchikov G. (2009). Did the Toba volcanic eruption of ~74 ka B.P. Produce widespread glaciation? J. Geophys. Res. 114 10.1029/2008JD011652

[B51] Schlecht J. Kaplan M. E. Barnard K. Karafet T. Hammer M. F. Merchant N. C. (2008). Machine-learning approaches for classifying haplogroup from Y chromosome STR Data. PLoS Comput. Biol. 4:e1000093. 10.1371/journal.pcbi.1000093 18551166 PMC2396484

[B52] Sengupta S. Zhivotovsky L. A. King R. Mehdi S. Q. Edmonds C. A. Chow C.-E.. (2006). Polarity and temporality of high-resolution Y-Chromosome distributions in India identify both indigenous and exogenous expansions and reveal minor genetic influence of central Asian pastoralists. Am. J. Hum. Genet. 78, 202–221. 10.1086/499411 16400607 PMC1380230

[B53] Smith V. A. (1921). The Oxford History of India: From the Earliest Times to the End of 1911. London: Oxford at the Clarendon Press.

[B54] Smolenyak M. S. Turner A. (2004). Trace Your Roots with DNA: Using Genetic Tests to Explore Your Family Tree. Emmaus, PA: Rodale.

[B55] Stringer C. (2000). Palaeoanthropology. Coasting out of Africa. Nature 405, 24–27. 10.1038/35011166 10811201

[B56] Subramanian N. Khan A. (2016). Who were the people of the Indus Valley Civilisation? The Indian Express (Accessed January 10, 2017).

[B57] Tan N. (2012). Prehistoric Skeleton Found in Sri Lanka. Available online at: https://www.southeastasianarchaeology.com/2012/06/27/prehistoric-skeleton-found-in-sri-lanka/ (Accessed July 3, 2016).

[B58] Vadivelu M. K. (2016). Emergence of sociocultural norms restricting intermarriage in large social strata (endogamy) coincides with foreign invasions of India. Proc. Natl. Acad. Sci. U.S.A. 113, E2215–E2217. 10.1073/pnas.1602697113 27071121 PMC4843417

[B59] Walter R. C. Buffler R. T. Bruggemann J. H. Guillaume M. M. Berhe S. M. Negassi B.. (2000). Early human occupation of the Red Sea coast of Eritrea during the last interglacial. Nature 405, 65–69. 10.1038/35011048 10811218

[B60] Wells S. (2007). Deep Ancestry: Inside the Genographic Project. Washington, DC: National Geographic Society.

[B61] Wood M. (2007). India. New York, NY: Basic Books.

[B62] Xing J. Watkins W. S. Hu Y. Huff C. D. Sabo A. Muzny D. M.. (2010). Genetic diversity in India and the inference of Eurasian population expansion. Genome Biol. 11:R113. 10.1186/gb-2010-11-11-r113 21106085 PMC3156952

[B63] Yaqoob M. Gustavson K. H. Jalil F. Karlberg J. Iselius L. (1993). Early child health in Lahore, Pakistan: II. Inbreeding. Acta Paediatr. Suppl. 82(Suppl. 390), 17–26. 10.1111/j.1651-2227.1993.tb12903.x 8219463

[B64] Zhao Z. Khan F. Borkar M. Herrera R. Agrawal S. (2008). Presence of three different paternal lineages among North Indians: a study of 560 Y chromosomes. Ann. Hum. Biol. 36, 46–59. 10.1080/03014460802558522 19058044 PMC2755252

